# Changes in the morphology traits, anatomical structure of the leaves and transcriptome in *Lycium barbarum* L. under salt stress

**DOI:** 10.3389/fpls.2023.1090366

**Published:** 2023-02-20

**Authors:** Xiao-Cui Yao, Li-Fang Meng, Wang-Li Zhao, Gui-Lian Mao

**Affiliations:** School of Life Sciences, Ningxia University, Yinchuan, China

**Keywords:** *Lycium barbarum* L., salt stress, expansin, transcriptome, leaf thickness

## Abstract

Salt stress directly affects the growth of plants. The limitation of leaf grow is among the earliest visible effects of salt stress. However, the regulation mechanism of salt treatments on leaf shape has not been fully elucidated. We measured the morphological traits and anatomical structure. In combination with transcriptome analysis, we analyzed differentially expressed genes (DEGs) and verified the RNA-seq data by qRT-PCR. Finally, we analyzed correlation between leaf microstructure parameters and expansin genes. We show that the leaf thickness, the width, and the leaf length significantly increased at elevated salt concentrations after salt stress for 7 days. Low salt mainly promoted the increase in leaves length and width, but high salt concentration accelerated the leaf thickness. The anatomical structure results indicated that palisade mesophyll tissues contribute more to leaf thickness than spongy mesophyll tissues, which possibly contributed to the increase in leaf expansion and thickness. Moreover, a total of 3,572 DEGs were identified by RNA-seq. Notably, six of the DEGs among 92 identified genes concentrated on cell wall synthesis or modification were involved in cell wall loosening proteins. More importantly, we demonstrated that there was a strong positive correlation between the upregulated *EXLA2* gene and the thickness of the palisade tissue in *L. barbarum* leaves. These results suggested that salt stress possibly induced the expression of *EXLA2* gene, which in turn increased the thickness of *L. barbarum* leaves by promoting the longitudinal expansion of cells of the palisade tissue. This study lays a solid knowledge for revealing the underlying molecular mechanisms of leaf thickening in *L. barbarum* in response to salt stresses.

## 1 Introduction

Soil salinization is a global environmental problem affecting the composition of ecosystems. The increasing number of salinized soils is a consequence of salinity in global warming, the evaporation rate, and over-cultivation, which has become a serious problem (Tang et al., 2015). This phenomenon of soil salinity is gradually aggravated in the future, as a result of poor-quality irrigation water, over-applicated fertilizer, and so on. Therefore, the salt tolerance mechanism of plants, especially halophytes, faced with salt is becoming imperative to elucidate at the morphology, physiology, biochemistry, and molecular level (Lv et al., 2019). Plant leaf, as the main vegetative organ for photosynthesis and transpiration, shows one of the earliest visible effects of salinity. The restriction of leaf growth is usually considered as an important parameter to evaluate plant tolerance to salt stress (Chen et al, 2014; Avestan et al., 2021). Leaf structure is the key to evaluate leaf growth. Navarro et al. observed that the anatomical changes in *Arbutus unedo* leaves under salt stress (0, 52, and 105 mM NaCl) showed that the cell size of the second layer of palisade cells significantly increased in parallel with the levels of salinity compared with control plants (Navarro et al., 2007). Leaf growth is affected by the time and space of expansion and mesophyll cell division. Moreover, leaf thickness increases with leaf expansion, along with changes in the size and longitudinal number of mesophyll cells (Hu et al., 2022). Another research found that the leaves of cucumber seedlings became smaller and thicker in response to salt stress (Yan et al., 2020). Meanwhile, the thickness of palisade tissue, sponge tissue, and the leaf structure became loose and disordered, and the intercellular space of mesophylls was thinner under salt stress. This would be explained by salt stress promoting cell division but inhibiting lateral expansion of leaf cells (Yuan et al., 2015). As for halophytes, the rapid thickening of the leaves has been often associated with a delay in the development, but longer survival of individual leaves under saline conditions (Zeng et al., 2018). However, the precise mechanisms of leaf thickening are still understood much less.

According to the “acid growth” theory, all authors are at a consensus that auxin-mediated acidification of the leaf apoplast induces leaf growth, which results in increased cell wall extensibility (Rayle and Cleland, 1970; Neves-Piestun and Bernstein, 2001; Spartz et al., 2016; Höfte and Voxeur, 2017; Polak and Karcz, 2021). The pH around the growing plant cells spans from 4.0 to 5.5, which is also the range in which acidification activates expansion activity (Geilfus and Mühling, 2011; 2012; Cosgrove, 2017). It is noteworthy that the plant cell wall will produce appropriate responses to adapt to salt stress. In order to tolerate salt stress, the plant can alter the expression of expansin genes in the cell wall. Expansins, as one of the important factors in mediating acid-induced leaf growth, are proteins of the four subfamilies, including α-expansin (EXPA), β-expansin (EXPB), expansin-like A (EXLA), and expansin-like B (EXLB), located in the cell wall (Marowa et al., 2016). They are involved in regulating plant abiotic stress tolerance and development by facilitating cell wall expansion in a pH-dependent manner (Chen et al., 2018). In addition, expansin proteins are involved in various developmental processes such as root growth (Liu et al, 2021b; Wang et al., 2019), plant growth (Li et al., 2022a; Marowa et al., 2020), leaf growth (İncili et al., 2022), stem growth (Santiago et al., 2018), stomatal aperture regulation (Wei et al., 2011), fruit ripening and softening (Valenzuela-Riffo et al., 2020; Chen et al., 2022), and salt stress response (Chalekaei et al., 2021; İncili et al., 2022). The activity of expansin correlates with the mRNA level of the target genes, and the transcription of expansin is regulated by the growth environment (Lee and Kende, 2001; Kuluev et al., 2017; Mu et al., 2021). Although expansin was reported to respond to abiotic stress in various species, it regulates the leaf structure of *L. barbarum* to coordinate with the leaf expansion and leaf thickening under salt stress is yet unclear.

*Lycium barbarum* L. is the only halophytic plant of the *Lycium* group in Solanaceae family. As a saline plant, *L. barbarum* exhibits remarkable adaptability for cultivation in saline and non-salinized soils (Li et al., 2022b). Previous studies in Ningxia *L. barbarum* have focused on ion transport and homeostasis, osmotic regulation (Yuan et al., 2016; Li et al., 2022b), and photosynthesis (Ma et al., 2021). However, the physiological and molecular mechanism behind its leaf morphological changes is still elusive. The aim of this study was to characterize the mechanism of leaf growth response to salt treatment in *L. barbarum*. The growth and microstructural and transcriptomic changes in the leaves of *L. barbarum* seedlings in response to different concentrations of NaCl stress for 7 days were investigated. This study improves our understanding of salt tolerance mechanism in *L. barbarum* leaves and provide an important information to explore the measurement for improvements of salt–alkali lands.

## 2 Materials and methods

### 2.1 Plant material, growth conditions, and salt treatment

The seeds of *L. barbarum* L. (Cultivar Ningqi 10) were immersed into 1% gibberellin for 30 min, followed by 10 min of disinfection with 0.1% potassium permanganate, then were grown in soil (nutrient soil:perlite:vermiculite = 3:1:1) in an artificial climate chamber (16 h light at 24°C/6 h night at 20°C). After the seedlings had grown two cotyledons, they were transferred to Hoagland’s nutrient solution for hydroponic growth; solutions were refreshed every 3 days. When seedlings reached the 10-leaf stage, salt stress was started by adding different concentrations of NaCl (0, 100, and 200 mmol·L^−1^) to the nutrient solution for additional 7 days. Leaves for transcriptome analysis and RNA isolation were frozen in liquid nitrogen and stored at −80°C.

### 2.2 Leaf microscopic analysis

Five replicate leaves of each treatment were selected to measure leaf thickness, leaf length, and leaf width after 7 days of different NaCl treatments. For anatomical characterization, 1–1.5-mm-thick leaves containing main veins were cut and collected and fixed overnight in 50% formalin–acetic acid–alcohol (FAA) solution as previously described (Moll et al., 2002). The leaf segments were dehydrated with a graded alcohol concentrations and embedded in paraffin. The sections were cut at 10 µm using an optical microtome, stained with toluidine blue for 2–5 min, rinsed with distilled water, and then placed in xylene for 10 min. The leaf microstructure was examined with a microscope. Additionally, leaf thickness was quantified with a microscope (Nikon Eclipse E100, JPN) using a calibrated scale bar provided by CaseViewer software.

### 2.3 Determination of leaf water content and water retention

*Lycium barbarum* leaves treated with salt for 7 days were rinsed with distilled water, quickly drained with surface water and weighed (FW), then dried to a constant weight (DW) in an oven at 65°C. The water content and dry fresh weight specific gravity of the leaves were calculated by the following equations:

Water content % = (FW − DW)/FW_0_ × 100%

Dry fresh weight specific gravity = FW/DW

The water-retaining capacity of the leaves was measured by weighing the initial fresh weight of the leaf (FW_0_), then placing it on a filter paper and measuring the fresh weight (FW_0_); then, the fresh weight (FW) was first measured every 2 min to every 20 min at the next 10 min for 2 h and constantly weighed (DW) in a blast furnace at 65°C. The water retention capacity was evaluated by the following formula:

Water-retaining capacity (WRC)% = (FW-DW)/(FW_0_-DW) × 100%

### 2.4 RNA extraction and transcriptome sequencing

Total RNA was extracted from plant root samples of *L. barbarum* treated with 0, 100, and 200 mM NaCl solution using column RNA isolation kit (Qiagen, Hilden, Germany) according to the manufacturer’s protocol. The RNA quality and integrity were analyzed using a NanoDrop 2000 Ultra-microspectrophotometer (Agilent Technologies, Santa Clara, CA, USA). Additionally, sequencing libraries were generated using NEBNext^®^Ultra™ RNA Library Prep Kit for Illumina^®^ (NEB, USA) following the manufacturer’s recommendations, and index codes were added to attribute sequences to each sample. The library preparations were sequenced on an Illumina Hiseq 2000 platform based on the manufacturer’s instructions. The sequence data have been submitted to the NCBI Sequence Read Archive (https://www.ncbi.nlm.nih.gov/sra) under BioProject accession number PRJNA902940.

### 2.5 Transcriptome *de novo* assembly

The RNA sample of each repeat was separately sequenced. Clean data were acquired by removing spliced sequences, low-quality reads, and erroneous reads, which was assembled using Trinity software to obtain unigene sequences. *De novo* assembly and comprehensive sequence library construction were carried out with pooled clean datasets of nine samples. DIAMOND software was used to compare unigene sequences to COG, GO, KEGG, KOG, Pfam, Swiss-Prot, TrEMBL, eggnog, and NR databases, finding proteins with the highest sequence similarity to a given transcript and searching for their functional annotation. A typical cutoff e-value was set <1.0×10^−5^. Unigenes for the final annotation information was obtained using the HMMER parameter E-value ≤ 1e^−10^, and BLAST parameter E-value ≤ 1e^−5^ GO annotations were performed using the BLAST2GO (http://www.geneontology.org/) program.

### 2.6 DEGs analysis and functional enrichment

To assay differentially expressed genes (DEGs) between different samples, the transcripts per million (TPM) reads method was used to calculate the expression level of each transcript. Quantification of gene abundance was made with RSEM (http://deweylab.biostat.wisc.edu/rsem/). DEG analysis was performed using DESeq2 (http://www.bioconductor.org/packages/release/bioc/html/DESeq.html) software with Q-values ≤ 0.05, where DEGs with |log2FC|>1 and Q-values ≤ 0.05 were considered as significant. There were three test groups, namely, G0 (A1_A2_A3_versus_B1_B2_B3), G1 (A1_A2_A3_versus_C1_C2_C3), and G2(B1_B2_B3_versus_C1_C2_C3), that we set. In addition, functional enrichment analysis was performed on DEGs, including GO functional enrichment analysis and KEGG pathway analysis. The metabolic pathways that met this threshold were defined as those significantly enriched in DEGs, using a threshold of Q-value < 0.05 in GO terms and metabolic pathways compared to the whole transcriptome.

### 2.7 DEG validation by qRT-PCR analysis

Total RNA was extracted from plant roots under control and salt stress conditions using the RNAeasy Plant Mini Kit (Qiagen, Hilden, Germany). RNA purity and concentration were analyzed using a Nano Drop 2000 ultra-microspectrophotometer (Agilent Technologies, Santa Clara, CA, USA). cDNA was reversed by using TIAN Script II cDNA (Tian Gen, CHN). The quantitative real-time PCR (qRT-PCR) was performed using SYBR Green qPCR SuperMix (Tian Gen,CHN). The constitutively expressed gene actin of *L. barbarum* was used as the interreference. Primer Express 3.0 (Premier Biosoft, USA) software was used to design the primer sequences for fluorescence quantitative analysis of actin and genes involved in expansion proteins (**Table 1**). Each experiment was repeated at least three times, and the reaction conditions were 95°C for 3 min and 39 cycles at 95°C for 10 s, 58°C for 20s, and 72°C for 30 s. The 2^−ΔΔCt^ method was used to calculate the relative expression of differential genes.

**Table 1:** Primers used for quantitative real-time PCR analysis.

Primers	Sequence (5’→3’)	*Tm* (°C)	Purpose
*EXLA2-F*	TTTCTAGTGCTTCTGCCCTTCAG	59	qRT-PCR analysis
*EXLA2-R*	AAAGCCGATTGCTAAATTTCCA	59	
*EXPA8-F*	CCTCTCCAGCACTTTGATTTAGC	58	
*EXPA8-R*	TCCAGCTTTGTATTTGGCAATTT	59	
*EXPA3-F*	TGGCAATCAAACGCAGTCTTA	58	
*EXPA3-R*	GAGCGATGGTCACTAGCTTTGAC	59	
*EXPA15-F*	TGGTCACAGCTACCAATTTTTGC	60	
*EXPA15-R*	CACCAGCCCCCTGCTTTAT	59	
*EXPA16-F*	CCTAACGACAATGGAGGATGGT	59	
*EXPA1f 6-R*	GGCATGGCAAGGTCGAAA	59	
*EXPA10-F*	TGGCAAAACAACGCTTACCTT	58	
*EXPA10-R*	CCATCGCCTGTGGTAACCTT	59	
*Actin-F*	CTCACTGAAGCACCTCTC	51	Reference genes
*Actin-R*	ACGACCACTAGCATACAAG	51	

### 2.8 Statistical analysis

All data were replicated at least three times, presented as mean ± standard error (SE), and analyzed by IBM SPSS Statistics 24 software, followed by Duncan’s multiple range test at 0.05 and 0.01 level. One-way analysis of variance (ANOVA) was used for comparison between groups.

## 3 Results

### 3.1 Salinity stresses promoted the growth of leaf

**Figure 1:**
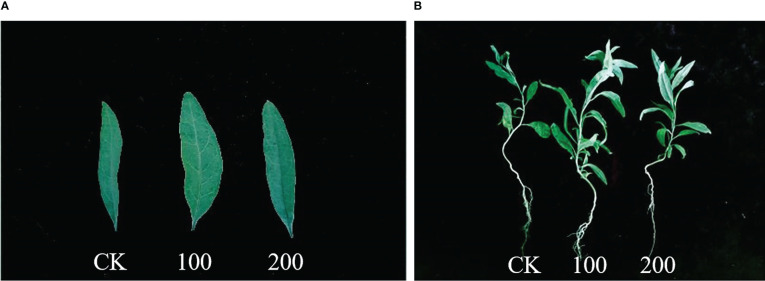
Phenotype of seedlings treated with different concentrations of salt for 7 days. **(A)** Leaf blade phenotype. **(B)** Whole plant phenotype. CK, 0 mmol·L^−1^. 100, 100 mmol·L^−1^. 200, 200 mmol·L^−1^.

**Figure 2:**
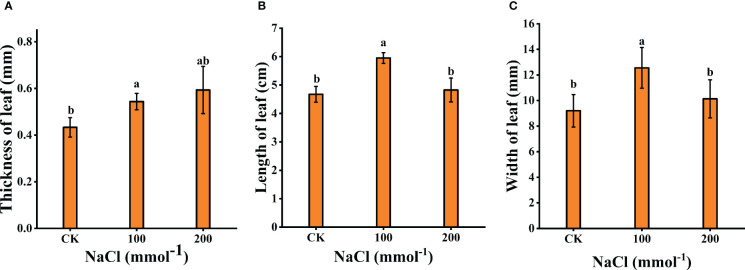
Effects of different salt concentrations on the growth of young seedlings in *Lycium barbarum* L. **(A)** Thickness of leaf. **(B)** Length of leaf. **(C)** Width of leaf. Data were analyzed by a Student’s t-test; a *p*<0.05 was considered as statistically significant.

Plant tolerance to salt stress was assessed by comparing changes in plant growth and leaf biomass (leaf thickness, leaf length, and leaf width). As shown in **Figures 1**, **2**, after 7 days of 100 mmol·L^−1^ NaCl, the leaves were visibly larger and healthier compared to the control group. The length, width, and thickness of leaves increased by 27.31%, 36.44%, and 11.54%, respectively. This shows that it has a stimulating effect on leaf lateral direction expansion under 100 mmol·L^−1^ NaCl. Notably, 200 mmol·L^−1^ NaCl insignificantly increased the leaf thickness (up to 26.97%, *p* < 0.05) rather than the length (3.13%, *p* < 0.05) and the width (9.19%, *p* < 0.05) of *L. barbarum* leaves compared with the control group.

### 3.2 Salt stress induced the alteration of water content and retention of leaf

**Figure 3:**
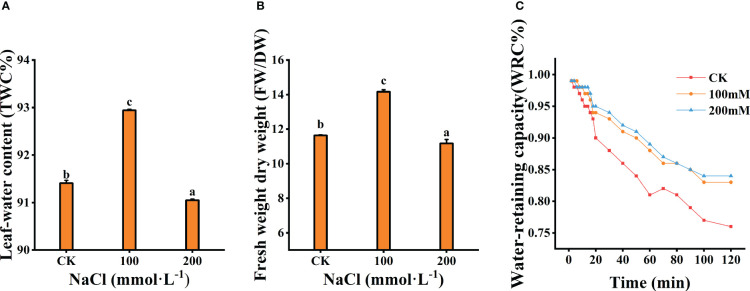
Effects of different salt concentrations on the water content and water-retaining capacity of the leaves. **(A)** Leaf-water content. **(B)** Fresh weight dry weight. **(C)** Water-retaining capacity. Data were analyzed by a Student’s t-test; a *p*<0.05 was considered as statistically significant.

The tissue water content (TWC) and the ratio of fresh weight to dry weight (FW/DW) of the leaves were measured after 7 days salt treatments. A total of 100 mmol·L^−1^ NaCl treatment significantly prompted the increase in TWC and the ratio of FW/DW (*p*<0.05), which increased by 36.44% and 21.71% compared to the control, respectively (**Figures 3A, B**). However, both decreased under 200 mmol·L^−1^ NaCl stress. Meanwhile, WRC of the leaf decreased with increasing treatment concentration (**Figure 3C**). The WRC with 200 mmol·L^−1^ NaCl was markedly lower than that of other treatments. These results indicate that moderate salt (100 mmol·L^−1^) could reduce the rate of water loss and increase the water retention of leaves.

### 3.3 Salinity stresses effected microstructure changes in leaf mesophyll cells

**Figure 4:**
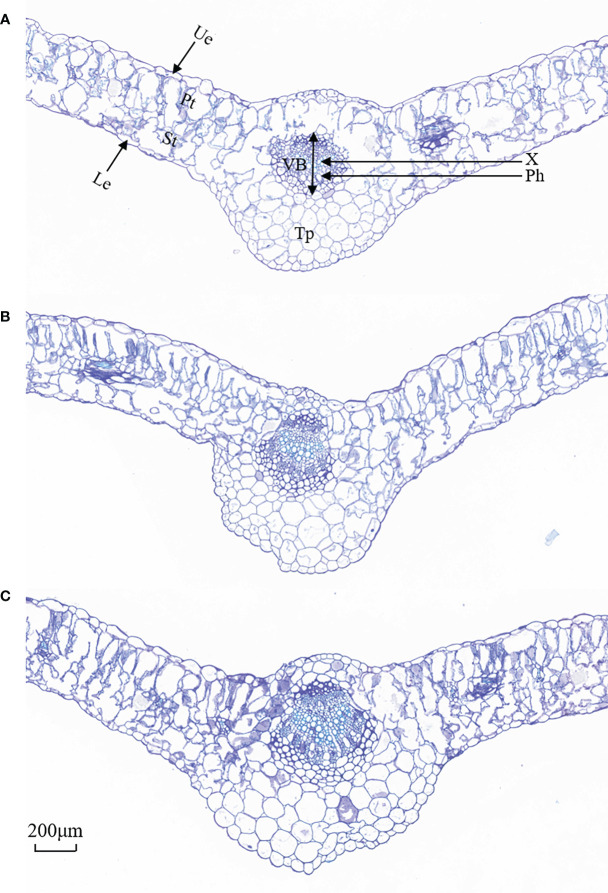
Representative images of cross-sectional structure of *Lycium barbarum* L. leaves (10×). **(A)** CK (0 mmol·L^−1^). **(B)** 100 mmol·L^−1^. **(C)** 200 mmol·L^−1^. Ue, upper epidermal; Le, lower epidermis; Pt, palisade tissue; St, spongy tissue; VB, vascular bundle; X, xylem; Ph, phloem; Tp, thick parenchyma (thick angles and parenchyma).

As shown in **Figure 4**, the upper and lower epidermis thicknesses were essentially affected when exposed to salt treatments. The tightly arranged mesophyll cells of *L. barbarum* leaf was found under salt stress. A larger cell density of the palisade tissue and a smaller cell size of the spongy tissue were observed. More striking was the difference in the palisade tissue; salt stress resulted in a large thickness increase especially in 200 mmol·L^−1^ NaCl treatment. Palisade tissue width was greatest at 100 mmol·L^−1^ NaCl (49.70% increased, compared with the control plants, *p*<0.05). Nonetheless, the spongy tissue thickness and palisade tissue width in salt stress showed first an increase (up to 52.56% and 49.70% under 100mmol·L^−1^ NaCl, compared with the control group, *p*<0.05) and then a decrease, which resulted in the highest ratio of palisade tissue and spongy tissue thickness (37.88%, *p*<0.05) adding 100 mmol·L^−1^ NaCl. The variation in CTR in salt treatment was consistent with that in the spongy tissue thickness. No significant difference in SR was observed under salt stress. These results indicate that NaCl stress has a significant effect on the epidermal and cellular tissue thickness of the leaves, especially 200 mmol·L^−1^ NaCl treatment. Likewise, salt stress altered the leaf vein anatomy. The main vein thickness and the vascular bundle thickness under salt stress were consistent with that in the spongy tissue thickness, increasing by 16.43% and 26.81% compared with that without salt treatment, respectively. Meanwhile, a significant increase was found in MV cell size, phloem thickness, xylem thickness, parenchyma thickness, and xylem to phloem thickness ratio with the increase in the concentration of NaCl (**Table 2**).

**Table 2:** Anatomical structure parameters of leaves treated with different concentrations of *Lycium barbarum* L..

Determining quota	CK	100 mmol·L^-1^	200 mmol·L^-1^
Ue thickness / (μm)	24.85±11.08 a	30.12±8.78 a	32.35±6.68 a
Le thickness / (μm)	14.77±4.02 ab	15.70±3.37 b	21.91±3.91 a
Leaf thickness / (μm)	279.26±13.58 b	279.67±7.89 b	307.63±15.25 a
Pt thickness / (μm)	126.87±35.84 b	134.88±11.05 ab	154.60±26.75 a
St thickness / (μm)	80.73±9.85 b	123.16±21.70 a	99.96±8.23 c
Pt / St	0.66±0.11 b	0.91±0.10 a	0.66±0.08 b
CTR / (%)	28.82±2.29 c	43.88±6.668 a	32.46±1.25 b
SR / (%)	44.98±10.61 a	48.18±2.88 a	49.99±6.48 a
MV thickness / (μm)	596.12±4.51 b	591.14±14.81 b	694.06±20.8 a
VB thickness / (μm)	159.52±21.68 b	158.92±8.86 b	202.28±20.06 a
Ph thickness / (μm)	43.62±9.37 b	59±3.53 a	64.48±4.32 a
X thickness / (μm)	38.5±2.48 c	55.36±4.29 b	76.28±19.92 a
TP thickness / (μm)	274.44±24.05 c	300.26±25.61 ab	325.92±22.07 a
X / Ph	0.9±0.13 c	0.94±0.11 ab	1.17±0.24 a

Ue, Upper epidermal. Le, Lower epidermis. Pt, Palisade tissue. St, Spongy tissue. CTR, Tightness. SR, Looseness. MV, Medium vein. VB, Vascular Bundle. Ph, Phloem. X, Xylem. Tp, Thick parenchyma (thick angles and parenchyma).

### 3.4 Transcriptome sequencing analysis

We selected three treatments with three biological replicates of *L. barbarum* leaves as material for RNA-seq sequencing analysis. After filtering and screening, clean data were obtained. We had performed sequence assembly with Trinity software. To reduce redundance in gene expression analysis, we saved only the longest transcript of each contig and defined them as unigenes. As shown in **Table 3**, a total of 90,489 unigenes were assembled, with an N50 of 1,493 unigenes. Among the assembled unigenes, approximately 38.58% ranged from 200 to 300 bp, whereas 23.61% ranged from 300 to 500 bp, and approximately 17.68% ranged from 500 to 1,000 bp. The unigenes between 1,000 and 2,000 bp accounted for 10.53% of all unigenes. The length of the other 9.59% of unigenes exceeded 2,000 bp. **Table 4** shows that 57.69 Gb of clean data was obtained, with each sample reaching 6.03 Gb of clean data. The percentage of Q30 base was more than 92.78%; the proportion of GC was approximately 43%. These results indicated that the sequencing quality was good for the next stage of analysis. The clean data from each sample was compared to the assembled Transcript or Unigene library. The mapped ratio of clean data to transcripts ranged from 77.15% to 77.97% **Table 5**. The result showed that the data were reliable and could be used for subsequent analysis.

**Table 3:** Statistical table of assembly results.

Demo Unigenes Length	Total Number	Percentage / (%)
200-300	34,910	38.58
300-500	21,367	23.61
500-1000	16,001	17.68
1000-2000	9,530	10.53
2000+	8,681	9.59
Total Number	90,489	
Total Length	69,653,536	
N50 Length	1,493	
Mean Length	7,697,458,918	

**Table 4:** Sequencing data of samples.

BMK-ID	Read Number	Base Number	GC Content / (%)	%≥Q30 / (%)
A1	21,648,604	6,471,704,060	43.25	93.82
A2	21,190,866	6,337,830,914	43.05	93.82
A3	21,234,550	6,347,845,140	43.09	94.09
B1	20,172,028	6,031,803,682	43.10	93.60
B2	21,043,998	6,294,939,350	43.34	94.01
B3	21,051,791	6,296,242,170	43.00	94.24
C1	21,956,133	6,566,957,692	42.81	94.06
C2	22,924,909	6,853,761,104	42.72	93.76
C3	21,692,405	6,486,272,634	42.78	92.78

**Table 5:** Comparison of sequencing data and assembly results.

BMK-ID	Clean Reads	Mapped Reads	Mapped Ratio/(%)
A1	21,648,604	16,845,746	77.81
A2	21,190,866	16,499,302	77.86
A3	21,234,550	16,554,069	77.96
B1	20,172,028	15,670,161	77.68
B2	21,043,998	16,409,029	77.97
B3	21,051,791	16,388,219	77.85
C1	21,956,133	16,939,895	77.15
C2	22,924,909	17,727,299	77.33
C3	21,692,405	16,833,582	77.60

**Figure 5:**
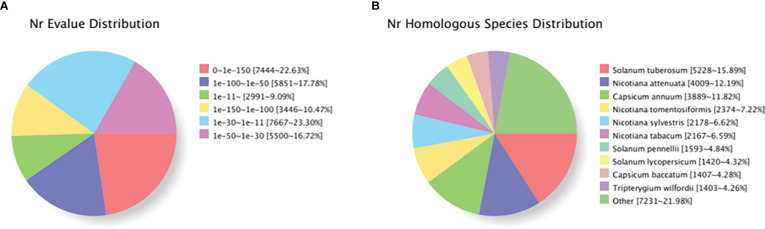
Characteristics of homology search of individual plants of *Lycium barbarum* L. using NCBI non-redundant (Nr) database. **(A)** Distribution of E-value for unique sequence blast hit with cutoff E-value of 1E^−5^. **(B)** Species distribution in each single-phase top BLAST with cutoff E-value of 1E^−5^.

Based on genes expression in each sample, the unigene sequence was compared with the Nr, Swiss-Prot, COG, KOG, eggNOG4.5, and KEGG databases using DIAMOND software, and the KEGG Orthotogy results were obtained using KOBAS. InterProScan used the database integrated with InterPro to analyze the GO orthogonal results of the new gene. After predicting the amino acid sequence of unigene, the annotation information of unigene was obtained compared with the Pfam database by the HMMER software. **Table 6** shows that TrEMBL and NR databases had the most unigenes with 32,966 (36.43%) and 32,899 (36.36%), respectively, followed by eggNOG database with 27,087 (29.93%), GO database with 25,592 (28.28%), Pfam database with 20,773 (22.96%), KEGG database with 20,621 (22.79%), and Swissprot database with 19,008 (21.01%), with the least being the KOG and COG databases with 17,075 (18.87%) and 7,530 (8.32%) unigenes, respectively. The distribution of annotated e-values based on the NR database showed that a large number of unigenes (50.88%) were less than 1E^−50^ (**Figure 5A**). When compared with the NR database, they were comparable to *Solanum tuberosum* (15.90%), *Nicotiana attenuata* (12.19%), *Nicotiana capsicum* (11.82%), *Nicotiana tomentosiformis* (7.22%), *Nicotiana sylvestris* (6.62%), *Nicotiana tabacum* (6.59%), *Solanum pennellii* (4.48%), *Solanum lycopersicum* (4.32%), *Capsicum baccatum* (4.28%), and *Tripterygium wilfordii* (4.26%), which had higher homology (**Figure 5B**).

**Table 6:** Numbers of annotated differentially expressed genes (DEG).

Anno_Database	Annotated_Number	300<=length<1000	length>=1000
COG_Annotation	7,530	1,663	4,634
GO_Annotation	25,592	8,161	12,098
KEGG_Annotation	20,621	6,222	10,555
KOG_Annotation	17,075	4,800	8,552
Pfam_Annotation	20,773	5,754	11,713
Swissprot_Annotation	19,008	5,516	10,376
TrEMBL_Annotation	32,966	11,179	14,943
eggNOG_Annotation	27,087	8,842	13,017
NR_Annotation	32,899	11,024	14,856
All_Annotated	34,988	11,770	15,030

**Figure 6:**
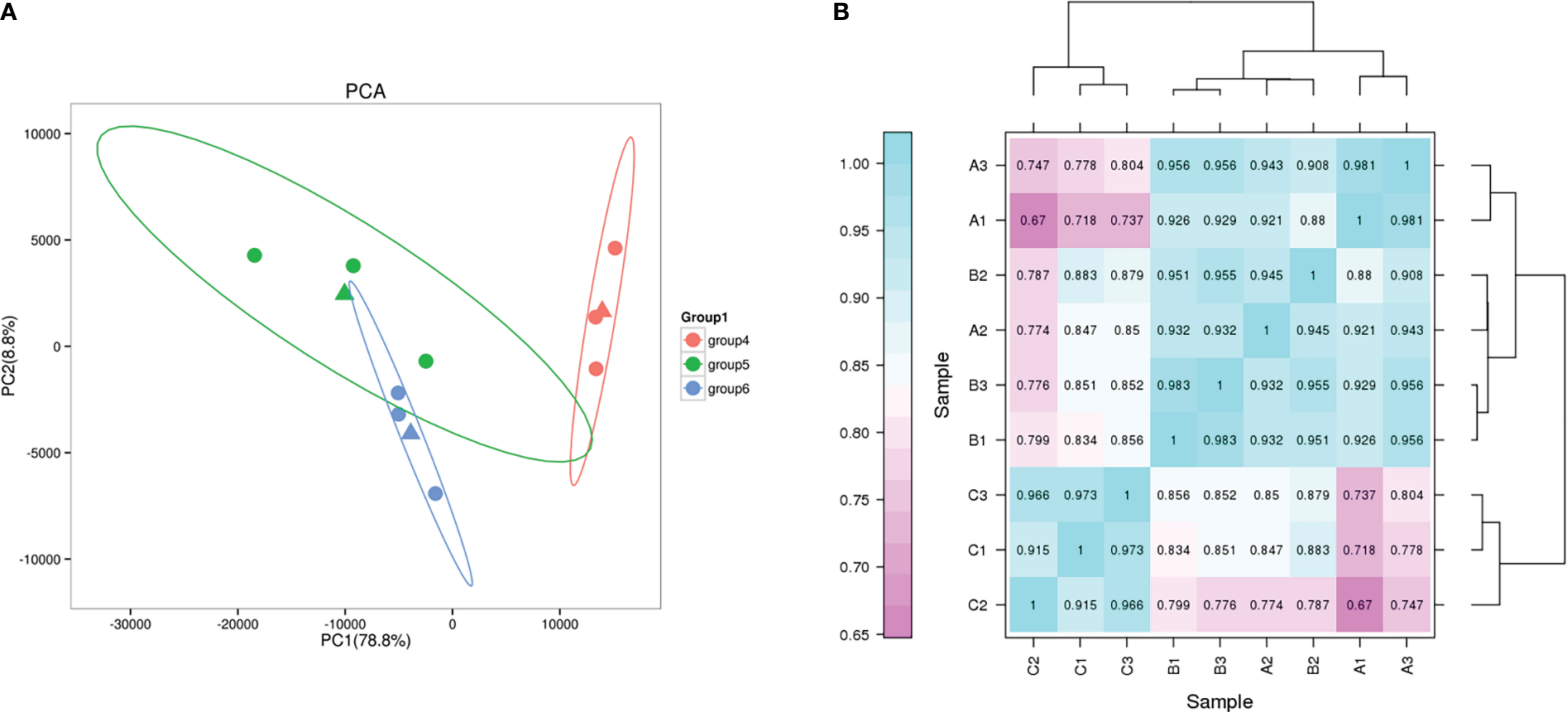
PCA and correlation analysis. **(A)** PCA plot of transcriptome profiles from different conditions. group4, G2; group5, G0; group6, G1. **(B)** Correlation analysis between different samples.

Principal component analysis (PCA) showed high similarity between biological replicates of the same group under different salt conditions (**Figure 6A**). Pearson correlation coefficient was used as an important indicator to evaluate the correlation between the gene expression in the nine samples. In this study, the biological replicates of the different treatments clustered together illustrate the reliability of the test (**Figure 6B**).

### 3.5 DEGs analysis and functional enrichment

**Figure 7:**
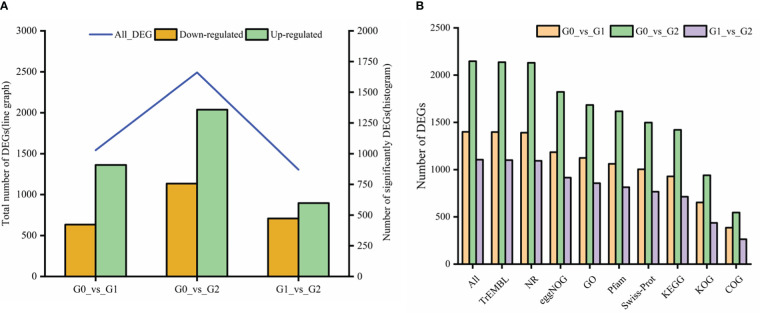
The number of differentially expressed genes (DEGs) and their functional annotations to seven public databases. **(A)** The number of DEGs were upregulated or downregulated in three groups. **(B)** All represent a total number of genes annotated in at least one the public database: TrEMBL, Translation of EMBL; NR, non-redundant nucleotide sequences; eggNOG, A database of orthologous groups of genes; GO, Gene Ontology; Pfam, Protein families; Swissprot, Swiss-Protein; KEGG, Kyoto Encyclopedia of Genes and Genomes; KOG, Eukaryotic Orthologous Groups and COG, Clusters of Orthologous Groups of proteins.

**Figure 8:**
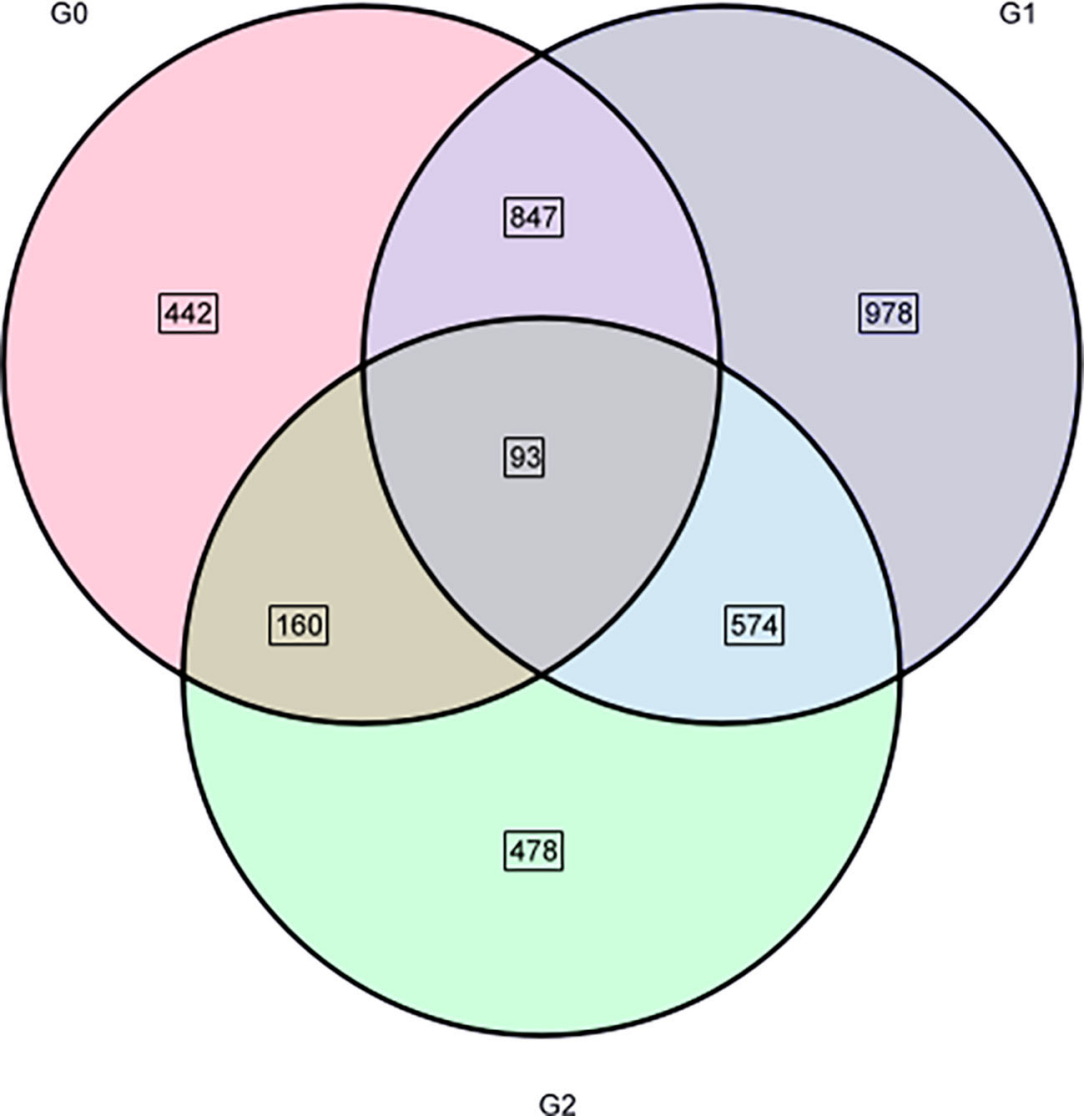
Venn diagram of the three groups of DEGs.

A total of 3,572 DEGs were identified with salt treatments and without treatment(log2FC) ≥ 1 and p-value < 0.05). Among them, 1,542 DEGs were detected containing 634 downregulated and 908 upregulated genes, respectively, in G0_*vs*_G1 (**Figure 7A**). In G0_*vs*_G2, there were 2,492 DEGs containing 1,134 downregulated and 1,358 upregulated genes. Of the DEGs (1305) in G1_*vs*_G2, 597 upregulated and 708 downregulated genes were identified. Compared with the control group, the number of up- and downregulated DEGs increased with increasing salt treatment concentration. Notably, total DEGs were successfully annotated into nine public databases; among them, TrEMBL and NR databases were the most involved in annotated DEGs (**Figure 7B**). **Figure 8** shows that a ternary Venn diagram was constructed based on a comparison of all DEGs in the salt treatment group with those in the control group, which had 442 unique DEGs in G0_*vs*_G1, 978 unique DEGs in G0_*vs*_G2, and 478 unique DEGs in G1_*vs*_G2. There are 93 DEGs in common among the three groups.

**Figure 9:**
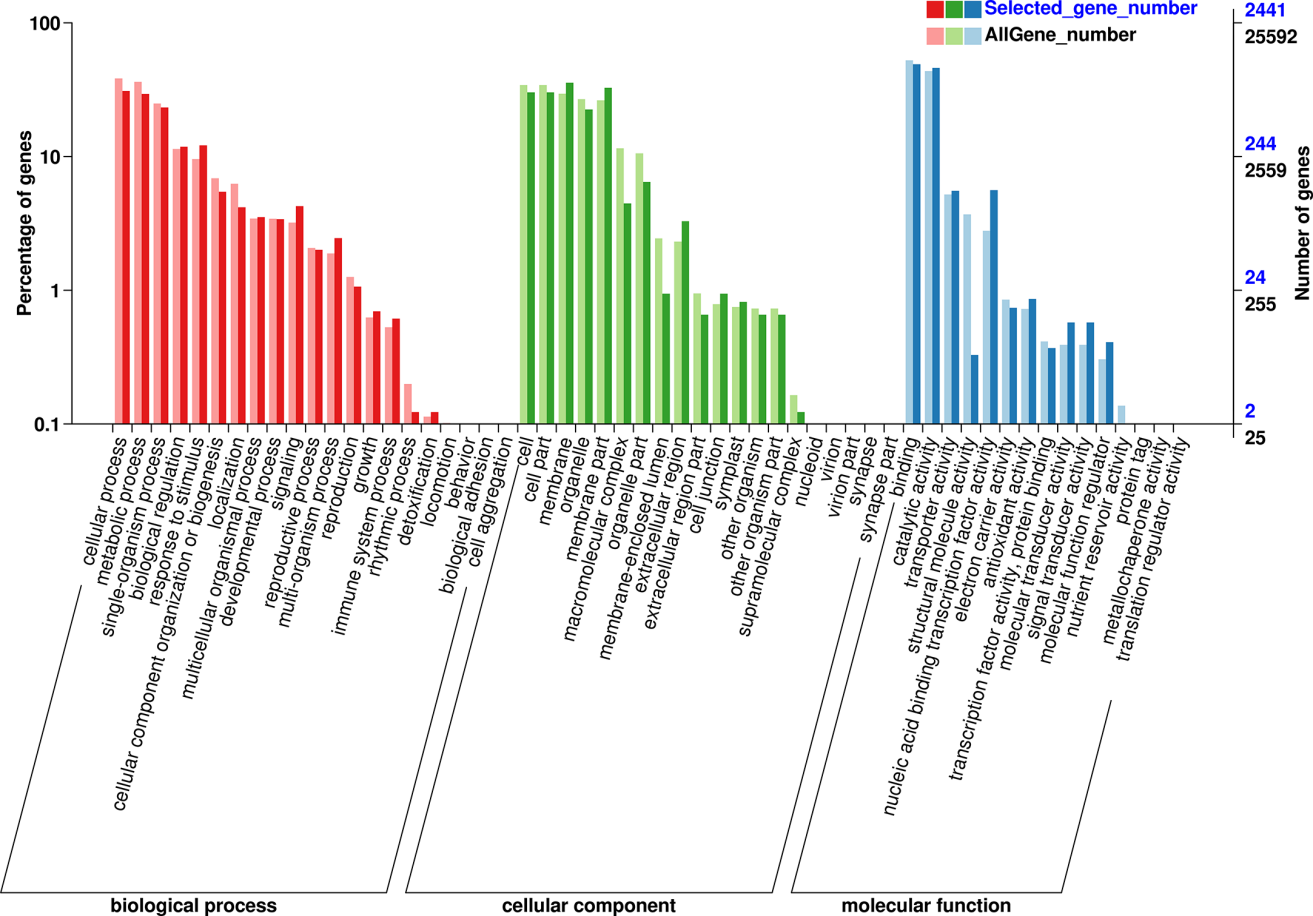
Gene ontology (GO) enrichment analysis of DEGs. GO classified as BP, CC, and MF.

**Figure 10:**
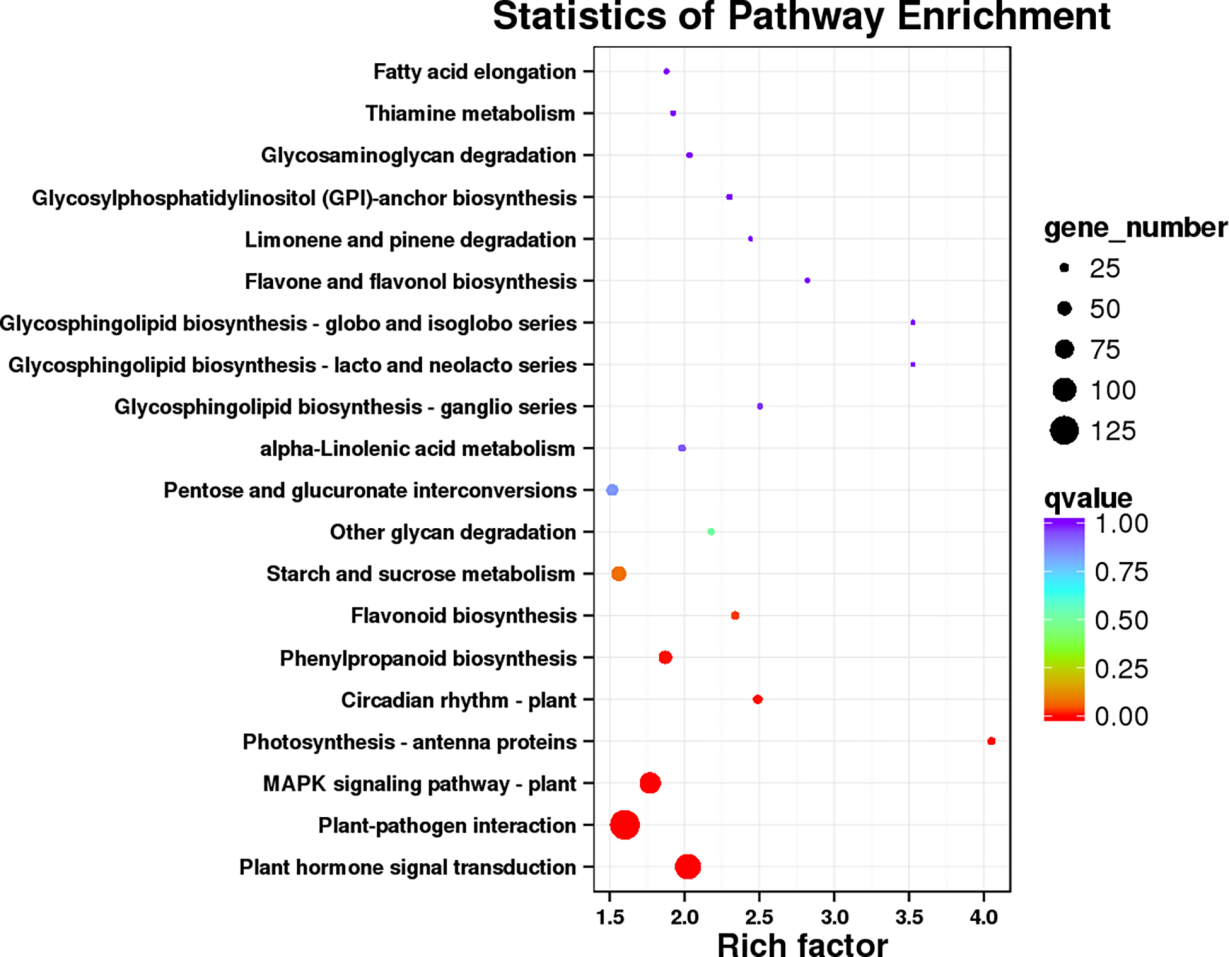
The database of KEGG enrichment analysis of DEGs.

GO and KEGG analysis were performed to obtained the functional formation of DEGs. The GO items were enriched into three types, namely, biological process (BP), cellular component (CC), and molecular function (MF). The BP was mainly enriched in metabolic process, cellular process, and single-organism process; the CC was mainly enriched in the cell, cell part, and membrane, while binding, catalytic activity, and nucleic acid binding transcription factor activity were mostly enriched in MF (**Figure 9**). KEGG enrichment analysis showed (**Figure 10**) that 1,016 DEGs were annotated to 127 metabolic pathways. The over-presented pathways were phytohormone signal transduction pathway, the plant pathogen interaction pathway, and plant MAPK signaling pathway.

### 3.6 Candidate of the cell-wall-related DEGs response to salt stress

A total of 92 DEGs related to cell wall synthesis or modification were identified (**Table 7**), including xyloglucan endotransglucosylase/hydrolase protein, cellulose synthase, and expansin, which were mostly upregulated in leaves stressed with 200 mmol·L^−1^ NaCl. Expansins, however, were downregulated in salt stress except for the upregulated expansin-like A2 (*EXLA2*) gene.

### 3.7 Validation of RNA-seq data by qRT-PCR

**Figure 11:**
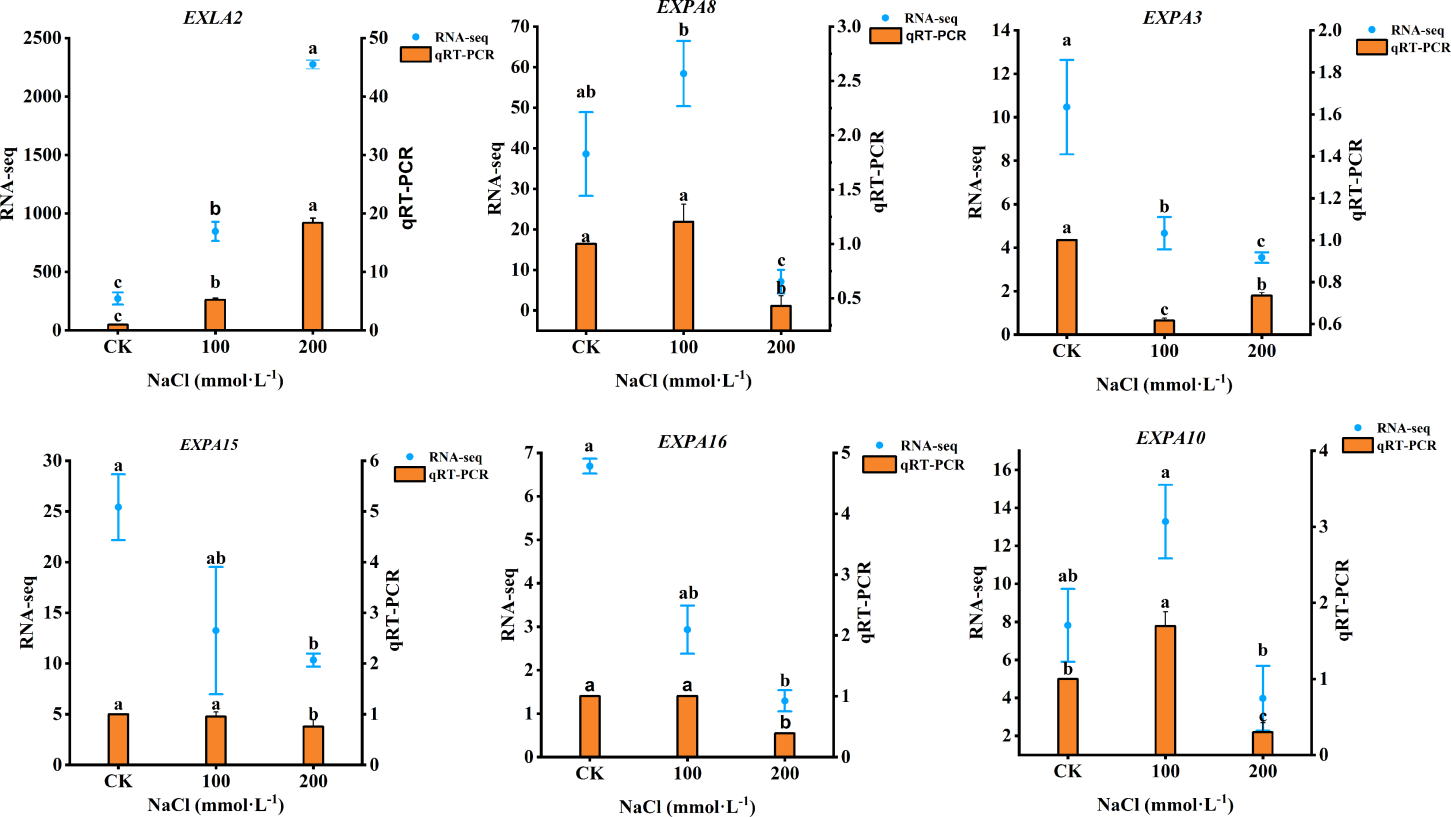
qRT-PCR results showing the mRNA expression level of six genes. Data were analyzed by a Student’s t-test; a *p*<0.05 was considered as statistically significant.

Based on the DEGs of plants under salt stress, six genes related to the expansin proteins (*EXLA2*, *EXPA8*, *EXPA3*, *EXPA15*, *EXPA16*, and *EXPA10*) were selected for quantitative RNA-Seq and qRT-PCR. As shown in **Figure 11**, *EXLA2* gene expression was upregulated both in 100 mmol·L^−1^ and 200 mmol·L^−1^ NaCl. The 100 mmol·L^−1^ NaCl induced the upregulated expression of *EXPA8* and *EXPA10* by increasing 20.37% and 69.48%, respectively (*p*< 0.05), but downregulated under 200 mmol·L^−1^ NaCl, which decreased by 57.04% and 70.12%, respectively (*p* < 0.05), whereas the expression of *EXPA3*, *EXPA15*, and *EXPA16* gene was a downregulated response to salt stress. The transcriptome data were in generally agreement with qRT-PCR, indicating that the RNA-seq data were valid and reliable.

### 3.8 Correlation analysis between leaf microstructure parameters and expansin genes

**Figure 12:**
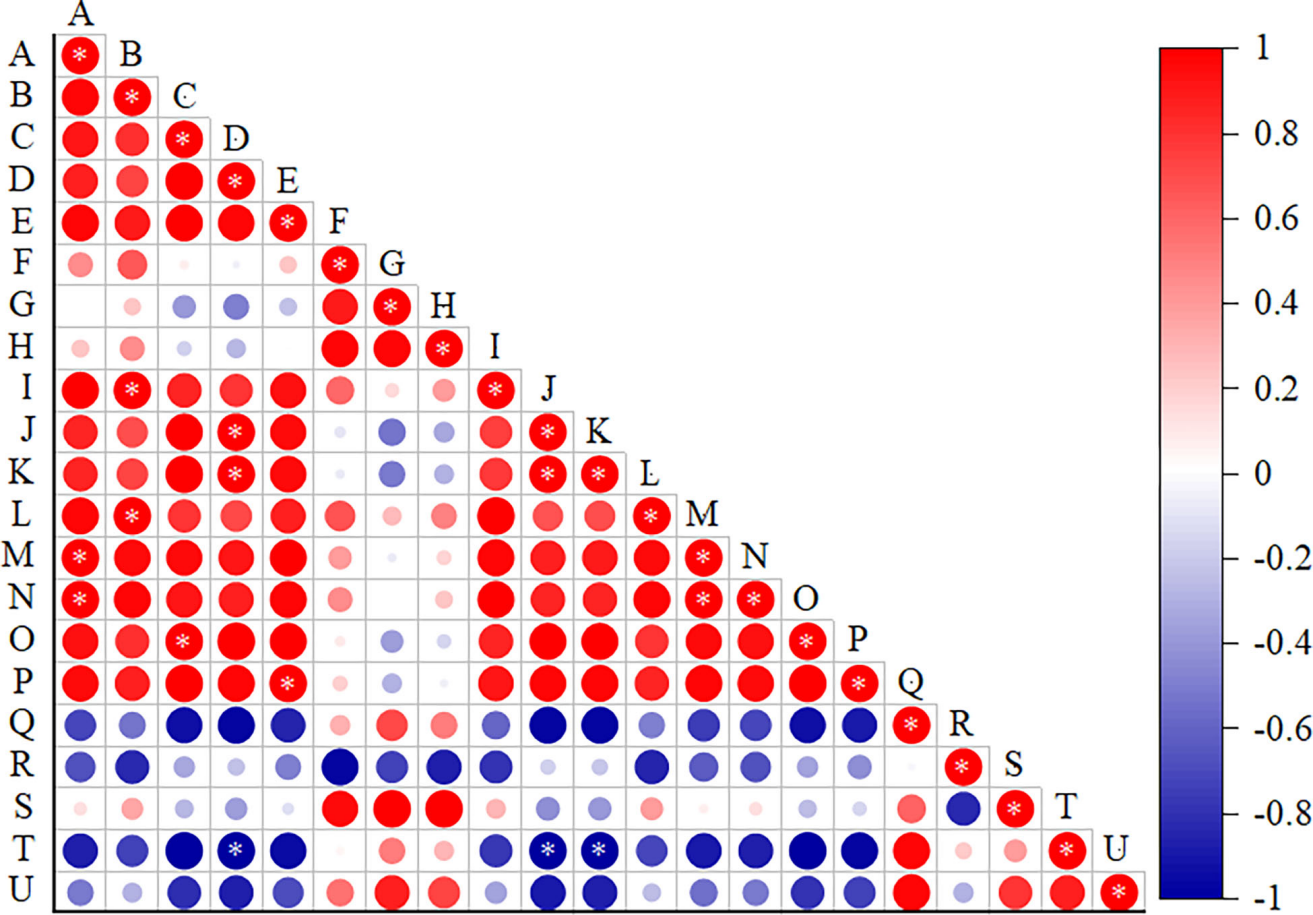
Correlation analysis of the leaf microstructural indicators and expansin genes. **(A)** Salt concentration, **(B)** Ue thickness, **(C)** Le thickness, **(D)** leaf thickness, **(E)** Pt thickness, **(F)** St thickness, **(G)** Pt/St, **(H)** CTR, **(I)** SR, **(J)** MV thickness, **(K)** VB thickness, **(L)** Ph thickness, **(M)** X thickness, **(N)** TP thickness, **(O)** X/Ph, and **(P–U)** expression levels of *EXLA2*, *EXPA8*, *EXPA3*, *EXPA15*, *EXPA16*, and *EXPA10*. Data were analyzed by a Student’s t-test; a *p*<0.05 was considered as statistically significant.

There was a positive relation between salt treatment concentration and xylem and thick parenchyma (*p*<0.05) between upper epidermal thickness and firmness of cell and phloem (*p*<0.05), between lower epidermal and the ratio of xylem to phloem (*p*<0.05), and between palisade tissue thickness and expression of *EXLA2* gene. There was a positive relationship between leaf thickness and main vein thickness and vascular bundle (*p*<0.05) but a negatively correlation with *EXPA16* gene expression (*p*<0.05) (**Figure 12**).

## 4 Discussion

Salt stress can result in the total physiological metabolic level changes in plants, which ultimately cause alternation of plant phenotype. The changes in morphological characteristics in responses to salt stress in plants are considered important for evaluating salt tolerance, of which leaves growth is the most (Zhao et al., 2021). However, how the leaf growth processes vary relative to the three length, width, and thickness of *L. barbarum* is rarely studied under salt stress. A study of two goji species found improved growth at a moderate salinity (100 and 200 mM NaCl) compared with the control (Li et al., 2022b). In this study, according to the phenotype growth of *L. barbarum*, we found that low concentrations of salt (100 mmol·L^−1^ NaCl) treatment significantly promote the leaves growth of *L. barbarum* by increasing the leaves length and width determined by cell division. Low concentrations of salt stress promote the leaf growth of *L. barbarum* mainly due to long-term adaptations to salt-stressed environments, which already have a variety of heritable adaptive traits that allow them to show some degree of resilience to salt-stressed environments and have developed a high tolerance to salinity (Mohammed et al., 2021; Palchetti et al., 2021). More importantly, leaves were thicker in high salt compared to low salt in contrast to the leaf length and width. As we all know, leaves development contains cell division and cell expansion processes (Gonzalez et al., 2012; Kalve et al., 2014), which contribute to differences in leaf area in response to salt stress. It indicated that low salt induced the width and length of *L. barbarum* leaves by promoting expansion rates in longitudinal and lateral direction (Kalve et al, 2014). As for plant leaf, a certain thickness is required and worth maintaining for whole plant functioning. In this study, the leaf thickness of *L. barbarum* gradually increased with increasing salt treatment concentration; especially, there was visible thickening on the leaf thickness of *L. barbarum*, and retention of the leaves was enhanced under 200 mmol·L^−1^ NaCl treatment. Many studies have indicated that the plant tissues with strong plasticity such as leaves first undergo morphological changes under salt stress (Gómez-Bellot et al., 2015; Acosta-Motos et al., 2015a; Acosta-Motos et al., 2015b). These plants generally retain water and improve water availability by leaf thickening under salt stress to improve their salt tolerance. In this study, the leaf thickness of *L. barbarum* gradually increased with increasing salt treatment concentration; especially, the leaf thickness of *L. barbarum* was visibly thickened, and retention of the leaves was enhanced under 200 mmol·L^−1^ NaCl treatment. To a certain extent, the thickening of the leaves reduces the evaporation of water from the leaves, and the water retention capacity of the leaves increases accordingly. These results indicated that *L. barbarum* could conserve water and prevent the water transport efficiency by increasing the ability of water storage under salt stress, which is considered as an important adaptive strategy to improve their salt tolerance to halophytes (Longstreth and Nobel, 1979; Zeng et al., 2018).

In the anatomical structure of *L. barbarum* leaves, we found that the thickness of the upper and lower epidermis is significantly increased, and there were closer and more neatly arranged epidermal cells under salt stress, which were consistent with the previous research in *Arbutus unedo* leaves (Navarro et al., 2007). The palisade mesophyll tissues contribute more to leaf thickness than spongy mesophyll tissues (**Table 2**). Large thickness of the palisade tissue was observed when NaCl was added; the increase in the thickness of the palisade tissue can promote the synthesis of organic matter to maintain the normal metabolism of plants, thereby reducing the damage caused by salt stress (Shen et al., 2022). Palisade tissue width in salt stress increased with 100 mol L^−1^ NaCl, which indicated that low salt facilitated cell expansion. In turn, cell expansion decided leaf growth (Gonzalez et al, 2012). It could be found that the thickening of leaves was caused by the longitudinal expansion of the palisade tissue and cells with salt application; the cells expanded toward the periphery with the leaf expansion. This was consistent with the research in which the thickening of the leaves was caused by the elongation of the palisade tissue in the response of *Myrtus communis* L. and *Eugenia myrtifolia* L. salt stress (Acosta-Motos et al., 2015a; Acosta-Motos et al., 2015b). It indicated that the leaf thickening of *L. barbarum* under salt stress was caused by the volume expansion of various cells in the leaves toward various dimensions. This phenomenon lays a structural foundation for the storage of nutrients in leaves to salt stress tolerance. In addition, salt stress also could produce different effects on plant leaf vein *via* the xylem area, which was reported in *Iperata cylindrica* (L.) Raeuschel as well (Hameed et al., 2009). The present study found that under the salt stress, the leaf vein thickness of *L. barbarum* increased in response to high salt treatment (200 mmol·L^−1^), indicating that *L. barbarum* could improve their ability to absorb water and nutrients and reduce water loss, thus increasing the relative water content of plants by increasing the thickness of phloem and xylem and the size of vascular bundles under salt stress (Li, 2022).

What is the underlying molecular mechanism of significant morphological changes in leaves by genes regulation in *L. barbarum* under salt stress? We identified 90,489 genes that were expressed among the leaves of *L. barbarum* through high-throughput Illumina sequencing, from which 3,572 DEGs were gained by GO enrichment; KEGG pathways enrichment analysis showed that salt stress induced the expression of 92 DEGs mostly upregulated and involved in cell wall synthesis or modification (**Table 7**). Plant cell walls are mainly composed of hemicellulose, cellulose, pectin, and so on (Kong et al, 2018). Among them, CESA is the key enzyme involved in cellulose synthesis (Turner and Kumar, 2018), xyloglucan and glucomannan in hemicellulose synthesis (Kumar et al., 2018), and PME and PAE in pectin synthesis (Kumar et al., 2018). Furthermore, XTH and expansin are key enzymes involved in cell wall loosening to promote cell elongation (Kumar et al., 2018). We found that most of these genes were unregulated, which protected plant cells from salt hurt *via* regulation of the metabolic process of the plant cell wall (CW) (**Table 7**). Previous studies have reported that salt stress has complicated effects on the expression of cell-wall-related genes (Qin et al., 2021). As a main component of cell wall, expansin plays a vital role in plant to abiotic stress tolerance by mediating cell wall expansion (Liu et al., 2021a; Sampedro and Cosgrove, 2005). In this study, we identified six DEGs related to expansin, and the expression of *EXLA2* gene was upregulated with the increase in salt stress. Abuqamar et al. determined the expression of *AtEXLA2* in response to salt stress. *AtEXLA2* was upregulated to salt, which indicates that plant responses to salinity stress can regulate *AtEXLA2* expression (Abuqamar et al., 2013). In addition, we hypothesized that the unregulated expression of *EXLA2* gene was related to the leaf thickening of *L. barbarum* in response to salt stress. It was observed in the microstructure that salt concentration promoted leaf thickening due to the enlargement of palisade tissue and cells, which associated with changes in the cell wall composition. To prove our hypothesis, in this regard, we correlated leaf microstructure with gene expression analysis of expansion. Correlation analysis showed that the expression of *EXLA2* gene was positively correlated with palisade tissue thickness. Previous studies showed that a certain concentration of salt stress can promote the activity of tonoplast H^+^-ATPase and plasma membrane H^+^-ATPase in *Arabidopsis thaliana* and *L. barbarum* cell membranes, acidify the cell wall by pumping out protons from the cell membrane, and promote the activation of expansin (Bulle et al., 2016). In this study, our hypothetical idea was further reinforced, namely, high expression of *EXLA2* gene induced response to salt stress, comparted excess salt ions into the cell wall with succulent tissue by proton pump H^+^-ATPase, promoted the thickness of palisade tissue and cells, which made thickened leaves to adapt to salt stress (Abuqamar et al., 2013). In addition, the other expansions including *EXPA3*, *EXPA8*, *EXPA15*, *EXPA16*, and *EXPA10* genes were all downregulated in response to 200 mmol·L^−1^ NaCl stress. It properly implied that high NaCl stress inhibited the expression of these genes, indicating that different expansin genes have different functions faced salt stress. These results will inform our further research into the potential molecular mechanisms of leaf thickening in *L. barbarum* in response to salt stress.

**Table 7:** Cell wall biosynthesis/modification related DEGs.

ID	G0_vs_G1	G0_vs_G2	G1_vs_G2	gene	family
c33306.graph_c0	normal	normal	down	XTH6	xyloglucan endotransglucosylase/hydrolase dsprotein
c41322.graph_c0	normal	Up	up	XTH15	
c51681.graph_c0	up	normal	normal	XTH8	
c56789.graph_c2	normal	Up	normal	XTH	
c59110.graph_c1	up	Up	normal	XTH	
c61562.graph_c0	up	Up	normal	XTH9	
c62703.graph_c1	up	Up	normal	XTH30	
c64658.graph_c0	normal	Up	up	XTH8	
c66853.graph_c0	up	Up	normal	XTH27	
c66916.graph_c1	up	Up	normal	XTH22	
c67790.graph_c1	up	normal	down	XTH27	
c68064.graph_c1	down	down	normal	XTH31	
c69153.graph_c0	normal	Up	up	XTH23	
c66690.graph_c2	normal	Up	up	XTH	
c66690.graph_c6	normal	Up	up	XTH	
c68053.graph_c3	normal	Up	up	CSLC4	Xyloglucan glycosyltransferase
c69088.graph_c0	up	Up	down	WAK2	Wall-associated receptor kinase
c48003.graph_c2	normal	Up	normal	GAE6	UDP-glucuronate 4-epimerase
c62137.graph_c0	up	normal	normal	GAE6
c66557.graph_c4	up	Up	normal	GAE6
c70710.graph_c0	normal	Up	normal	GAE1
c56198.graph_c1	normal	Up	normal	MUR4	UDP-arabinose 4-epimerase
c59089.graph_c0	normal	normal	down	GUX1	UDP-glucuronate
c62425.graph_c0	normal	Up	normal	UGD3	UDP-glucose 6-dehydrogenase
c64302.graph_c0	normal	Up	normal	UGD3	
c54084.graph_c0	normal	down	down	TBL28	trichome birefringence-like
c56452.graph_c1	normal	down	down	TBL36	
c62826.graph_c0	up	Up	normal	TBL39	
c41860.graph_c0	normal	normal	down	SUS6	Sucrose synthase
c41860.graph_c1	normal	normal	down	SUS6	
c69754.graph_c1	normal	down	normal	SBT3.4	Subtilisin-like
c69836.graph_c1	normal	down	down	LRK10	Rust resistance kinase
c58120.graph_c0	normal	down	down	At2g20870	Putative cell wall protein
c69469.graph_c1	up	normal	normal	PR5K	PR5-like receptor kinase
c68705.graph_c3	up	normal	normal	PGIP2	Polygalacturonase inhibitor
c41672.graph_c0	up	Up	normal	PME41	pectinesterase
c53737.graph_c0	down	normal	up	PECS-2.1	
c57833.graph_c0	normal	normal	down	PME29	
c62749.graph_c0	normal	normal	down	PME22	
c65008.graph_c2	up	normal	normal	PME	
c63704.graph_c0	up	normal	normal	PAE12	Pectin acetylesterase
c69320.graph_c1	up	normal	normal	PAE8	
c70062.graph_c0	down	down	normal	PAE11	
c53520.graph_c0	normal	normal	down	ERECTA	LRR receptor-like serine/threonine-protein kinase
c61237.graph_c1	normal	down	down	IRK	
c65748.graph_c2	up	normal	down	RGI3	
c65916.graph_c0	up	Up	normal	At1g74360	
c61801.graph_c0	normal	down	down	RPK2	
c68769.graph_c1	up	Up	normal	RGI3	
c69760.graph_c3	normal	Up	normal	At1g56140	
c62971.graph_c0	normal	down	normal	PXC1	Leucine-rich repeat receptor-like protein
c69459.graph_c5	up	Up	up	At5g35370	G-type lectin S-receptor-like serine/threonine-protein
c60817.graph_c0	up	Up	normal	RCOM_0530710	Glycosyl transferase family
c70219.graph_c0	up	normal	normal	GRP-1	Glycine-rich cell wall structural protein
c57498.graph_c0	normal	normal	down	CSLA2	Glucomannan 4-beta-mannosyltransferase
c57498.graph_c1	normal	normal	down	CSLA2	
c65626.graph_c4	up	Up	normal	CSLA9	
c68899.graph_c1	normal	normal	down	CSLA9	
c70541.graph_c0	normal	normal	down	CSLA9	
c50767.graph_c1	up	Up	normal	GATL9	Galacturonosyltransferase-like
c54030.graph_c0	up	Up	normal	GATL1	
c62099.graph_c0	normal	normal	down	GAUT12	Galacturonosyltransferase
c67662.graph_c0	normal	Up	normal	GAUT5	
c63141.graph_c0	up	Up	normal	FLA9	Fasciclin-like arabinogalactan protein
c49912.graph_c0	normal	normal	down	EXPA10	Expansin
c51480.graph_c0	normal	down	down	EXPA3	
c55782.graph_c0	normal	down	down	EXPA8	
c56797.graph_c0	normal	down	normal	EXPA3	
c59033.graph_c0	normal	down	--	EXPA1	
c59033.graph_c1	normal	down	down	EXPA15	
c60996.graph_c0	normal	down	--	EXPA15	
c68991.graph_c1	up	Up	normal	EXL2	EXORDIUM-like
c62854.graph_c1	normal	Up	normal	CHN48	Endochitinase
c41993.graph_c0	down	down	normal	DFRA	Dihydroflavonol 4-reductase
c54143.graph_c1	down	normal	normal	SNL6	Cinnamoyl-CoA reductase-like
c54042.graph_c0	normal	down	normal	CSP41B	Chloroplast stem-loop binding
c57123.graph_c0	normal	--	down	CSLD5	
c57123.graph_c1	up	--	down	CSLD5	
c62228.graph_c0	down	normal	normal	CSLG3	
c66029.graph_c0	normal	up	up	CSLD3	
c69291.graph_c0	normal	down	normal	CSLG2	
c69518.graph_c2	normal	up	normal	CSLE6	
c69518.graph_c3	normal	up	normal	CSLE6	
c69555.graph_c2	normal	up	up	CSLD3	
c59403.graph_c2	up	up	normal	CESA1	Cellulose synthase
c61874.graph_c0	normal	normal	down	CESA4	
c40836.graph_c0	down	normal	normal	C/VIF1	Cell wall / vacuolar inhibitor of fructosidase
c60331.graph_c0	normal	up	up	C/VIF2	
c64996.graph_c0	normal	normal	down	CDC2C	Cell division control protein
c63747.graph_c0	up	up	normal	CALS12	Callose synthase
c62512.graph_c0	normal	normal	down	IRX9	Beta-1,4-xylosyltransferase
c66442.graph_c2	normal	up	normal	RHM1	UDP-glucose 4,6-dehydratase

All the data in the table |log2FC| > 1 and Q value ≤0.05.

## 5 Conclusion

Low concentrations of salt (100 mmol·L^−1^NaCl) treatment significantly promote the leaves growth of *L. barbarum* by increasing the leaves length and width. In the country, high salt stress promoted the thickening of the leaves, increased leaves thickness, reduced water loss, and increased the water retention capacity of the leaves. The palisade mesophyll tissues contribute more to leaf expansion and thickness. Additionally, high salt treatment induced the thickness of leaves main vein, phloem and xylem thickness, vascular bundle size, and parenchyma thickness, which improve their ability to absorb water and nutrients and reduces water loss. Transcriptome analysis further revealed the expression of multiple expansin-related DEGs in *L. barbarum* leaves treated with different salt concentrations for 7 days. There were six expansin proteins that were screened; the expression of *EXLA2* gene was upregulated with the increase in salt stress, which promoted the expansion of palisade tissue cells, thereby increasing the leaf thickness. Therefore, further study is needed to define functions of *EXLA2* gene in the future. Our study thus provided information for further understanding the molecular mechanism and salt tolerance of *L. barbarum* by leaf thickening.

## Data availability statement

The original contributions presented in the study are publicly available. This data can be found here: NCBI, PRJNA902940.

## Author contributions

X-CY: conceptualization, formal analysis, data curation, investigation, methodology, software, and writing—original draft. L-FM: conceptualization, data curation, formal analysis, investigation, methodology, software, and writing—original draft. W-LZ: validation. G-LM: conceptualization, supervision, funding acquisition, project administration, resources, and writing—review and editing. All authors contributed to the article and approved the submitted version.
